# Cystitis

**Published:** 1894-11

**Authors:** E. T. Cook

**Affiliations:** President Houston District Medical Association; Surgeon in charge of St. Joseph (the County) Hospital; Houston


For Texas Medical Journal.

### CYSTITIS.

BY E. T. COOK, M. D.,
President Houston District Medical Association; Surgeon in charge of St. Joseph (the County) Hospital.

[Read before the Houston District Medical Association.]

IN CHOOSING the subject of "Cystitis" on which to write a paper, I have not done so with the view of furnishing for your edification a learned treatise on the subject, or a compilation of what authors of repute may have written about the matter, but rather have I tried to present briefly a few points that have come under my observation in the treatment of this almost incurable disease. The prime object of my paper will be to call the attention of the Association to the little success that is ordinarily attained by the methods of treatment now in vogue, and, if possible, invite a discussion of a subject in which I feel a great interest, and in the treatment of which I have by no means had satisfactory results. I have at times been almost constrained to place this disease along with our continued fevers, so far as the success of our methods was concerned. Here we go wildly to treating, getting generally bad results, occasionally good, but always *looking* wise, and invariably *feeling* profane.

As idiopathic cystitis is seldom met, save possibly in gouty subjects, I will divide the disease into acute and chronic. The acute form is characterized by local pain, a sense of weight about the hypogastric and iliac regions, tenderness on pressure, and extreme irritability about the bladder. There is more or less constitutional disturbance. When a small quantity of urine accumulates, an instantaneous desire to void the same occurs, and the act is accompanied with great tenesmus and intense suffering. The urine is highly colored, mixed with mucus or pus, and often tinged with blood. In my experience, I have seldom been able to cut short this form of cystitis, but find that it usually terminates in the chronic form of the disease, gradually undergoing resolution. The introduction of the catheter in acute cystitis, I think, is contraindicated, on account of the irritation produced thereby. I usually give mucilaginous drinks, to dilute the urine, and alkalies, such as bicarbonate of potash, etc., to render the urine less irritating, keeping the patient in bed and using hot poultices or fomentations applied to the lower part of the abdomen. Hot hip baths, persistently used, sometimes give great relief. Of course, if the urine becomes foul and fetid the catheter may be introduced and the bladder washed out, as the danger of retention of the foul urine will be greater than the harm arising from the passage of the catheter. The authorities cite numerous cases of fatal cystitis (acute), death being the result of septicæmia, suppurating kidney, and rarely, peritonitis. I have met no such cases.

Chronic cystitis, the form of the disease that is most commonly met,—and, by the way, have the gentlemen of the Society noticed how exceedingly common it is, and how often they meet it in their practice?—is the form of the disease with which I want to more particularly deal in this paper. The vast majority of cases I have met are the result of gonorrhœa and its sequalæ, the tight stricture. Gonorrhœa which has been neglected, and as a result has entailed on its victim a career of life-long misery. By way of parenthesis, let me say here that I believe the profession has been derelict in its duty to the public in not impressing on their patients the seriousness of an attack of gonorrhœa. I believe that gonorrhœa has entailed more suffering and misery on the human family than even syphilis, and chiefly because our young men have become imbued with the fallacious idea, as they facetiously express it, that "clap is no worse than a bad cold."

A few years ago the profession seemed to be a unit in favor of washing out the bladder in all cases of cystitis. This I followed for a number of years, with by no means satisfactory results. On the contrary, I found that instead of doing good I often did injury by throwing astringent antiseptic solutions in the bladder. The irritation caused by the passage of the catheter did more harm than good, and of late I never wash out the bladder except to relieve it of foul urine or to disinfect, when it becomes in my opinion necessary to do so.

The treatment of chronic cystitis must always have reference to its cause. If due to stone or stricture no permanent cure can be reasonably expected until these causes are removed.

To sum up, I have found the following course of treatment the most efficacious. Should decomposition of urine—and it most generally is—be a complication, the bladder should be washed out after having drawn off the accumulated urine by throwing into it about two ounces of lukewarm water containing enough permanganate of potash to make it the color of light claret. This can be repeated until the fluid is expelled without the admixture of urine or pus.

Along with this antiseptic local treatment the patient should be given a prescription about as follows:

℞ Balsam of copaib........................ ʒi.

Tr. cubebs............................... ℥ss.

Salol.................................... ʒi.

Syr. acaciæ q. s......................... ℥iv.

Teaspoonful three times a day.

Of course the diet should be regulated, and the patient kept quiet. I have found this to be possibly the most successful treatment I have used. Every physician however, has his favorite line of treatment. The various astringents antiseptics and cauterants are used. As before stated the specific cause must always be sought for, and if possible eliminated, but with me it has often been the case that after removal of the cause, as far as possible, the cystitis has remained indefinitely, finally involving the deeper structures of the bladder and resulting in cystirrhœa. These are the cases to which I have particularly referred in this paper, as having been in my hands almost incurable.

In connection with Dr. Dolen, I recently treated a case of chronic cystitis in St. Joseph's Hospital, Houston. It was very obstinate and would yield to nothing we could devise. If we did hit upon a treatment that seemed to relieve for the time, the least change in the weather would bring a recurrence, if anything, more severe than the last. I had some samples of coal oil emulsion which I prescribed and which did him great good, so he expressed. The samples gave out and he relapsed back into his former condition.

Being unable to secure any more of the samples, I put him on the prescription cited above, and he began at once to improve, continuing to do so until he was finally discharged about well. I am of the opinion, though, that on the least exposure he will have a recurrence with all symptoms as violent as ever. Apropos of the treatment of this disease, I will say that a few weeks ago I read in a medical journal of the treatment adopted by some surgeon of England. He entered the bladder above the pubes, threw in his antiseptic and astringent solution, and drained through the urethra. He reports great success by this treatment, but, of course, it could not be adopted in the general practice, from the fact that very few patients would submit to such radical treatment.

Now, if I have succeeded in merely causing the members of this society to think of this very common disease, and our inability to successfully treat it, to the extent that we will get their views and experience, I feel that the object of my paper has been attained. It is surprising, considering the seriousness of the disease, to see how deficient our literature is on the subject, most recent authors either ignoring it or passing it over as though they were afraid to deal with it.

